# Initial-Dip Existence and Estimation in Relation to DPF and Data Drift

**DOI:** 10.3389/fninf.2018.00096

**Published:** 2018-12-11

**Authors:** Muhammad A. Kamran, Malik M. Naeem Mannan, Myung-Yung Jeong

**Affiliations:** Department of Opto-Mechatronics Engineering, Pusan National University, Busan, South Korea

**Keywords:** functional near-infrared spectroscopy, initial dip, hemodynamic response, optimal cortical model, optical neuro-imaging

## Abstract

Early de-oxygenation (initial dip) is an indicator of the primal cortical activity source in functional neuro-imaging. In this study, initial dip's existence and its estimation in relation to the differential pathlength factor (DPF) and data drift were investigated in detail. An efficient algorithm for estimation of drift in fNIRS data is proposed. The results favor the shifting of the fNIRS signal to a transformed coordinate system to infer correct information. Additionally, in this study, the effect of the DPF on initial dip was comprehensively analyzed. Four different cases of initial dip existence were treated, and the resultant characteristics of the hemodynamic response function (HRF) for DPF variation corresponding to particular near-infrared (NIR) wavelengths were summarized. A unique neuro-activation model and its iterative optimization solution that can estimate drift in fNIRS data and determine the best possible fit of HRF with free parameters were developed and herein proposed. The results were verified on simulated data sets. The algorithm is applied to free available datasets in addition to six healthy subjects those were experimented using fNIRS and observations and analysis regarding shape of HRF were summarized as well. A comparison with standard GLM is also discussed and effects of activity strength parameters have also been analyzed.

## Introduction

Near-infrared spectroscopy (NIRS) is an emerging non-invasive neuro-imaging methodology that measures the cortical activity based on blood chromophores (Noori et al., 2017; Khan et al., 2018). It is well-known fact that regional blood flow and neural activities are tightly coupled in time and space (Lindauer et al., 2001; Salvador et al., 2010; Whiteman et al., 2017). Functional near-infrared spectroscopy (fNIRS), therefore, measures cerebral blood volume and blood oxygenation changes as indicators of neural activity (Talukdar et al., 2013). Continuous-wave near-infrared spectroscopy (CW-NIRS) determines the concentration changes of oxy-hemoglobin (Δ*HbO*) and deoxy-hemoglobin (Δ*HbR*) by shining, through the scalp, near-infrared (NIR) light of different wavelengths (~ 630–920 nm) into cortical tissue (Scholkmann and Wolf, 2013). The absorption and scattering of NIR light are characterizing features that formulates an estimate of HbO and HbR concentration change (Prakash et al., 2007; Kamran et al., 2015). The cortical information received by fNIRS relates to the local hemodynamic response, unlike electroencephalography, which can quantify electric brain activity in the field. The simultaneous recording from multiple locations on surface of scalp could result in improved accuracy and reliability. fNIRS has several advantages over other currently existing neuro-imaging modalities, which also measure hemodynamic response function (HRF) characteristics. Those includes reasonable spatial resolution and high temporal resolution suitable for brain-computer-interface (BCI) applications (Jasdzewski et al., 2003). The details on the advantages, limitations, and challenges in the field of fNIRS can be found in Kamran et al. (2015).

Initiating the neuronal activity cause oozing of HbO in blood flow (Buxton, 2001). Glucose, oxygen and other nutrients are major components in the blood to maintain healthy brain functioning (Buxton et al., 2004). For this, cerebral blood flow (CBF) increase is essential. Additionally, the CBF increase is required to carry out carbon dioxide and other waste from particular activated region (Buxton, 2001). The initial oxygen requirement or deoxygenating increase in a localized brain region is defined in the literature as the “initial dip” (Kamran et al., 2016). The existence of the initial dip is still a controversial issue but it could be incremental feature for effective BCI applications. The immediate detection/estimation of brain commanding signal is crucial step for such efficient, effective and fast BCI systems. One possibility is to achieve this by analyzing metabolic signal instead of blood volume related indication (Hong and Naseer, 2016). Therefore, initial dip has strong attraction to research community due to its metabolic relation. Some of the previous fMRI studies have reported the existence of early de-oxygenation prior to the initiation of oxy-hemoglobin rise (Menon et al., 1995; Yacoub and Hu, 2001). Similarly, fNIRS studies have reported the existence of an initial dip in some cases and experiments. Jasdzewski et al. (2003) observed that the initial dip exists as a part of the HRF and is caused by early de-oxygenation after presentation of brief stimuli. Menon et al. (1995) recorded an initial negative change in measured signals after onset of photic stimulation. Later, Hu et al. (1997) concluded that the early response could be selectively and reliably mapped in individual subjects. Additionally, to this, they observed that the characteristics of the early response's signal change were independent of stimulus duration for stimuli longer than 3 s. Mayhew et al. (2000) investigated the stimulation effects of the barrel cortex in anesthetized rats using NIR light spectroscopy. Their observations suggest the existence of HbR increase before HbO increase, though it was not as large as the main response. The evidence of initial dip against electrical vibrissae stimulation were presented in Jones et al. (2001). Moreover, several studies related to fNIRS have discussed and analyzed the detection and existence of initial dip in general case/BCI applications (Akiyama et al., 2006; Prakash et al., 2007; Yoshino and Kato, 2012; Hong and Naseer, 2016). Therefore, it is a paramount to dig out factors that can affect the existence/appearance of initial dip.

The radiant NIR light ravine through tissues, capillaries, and blood before a part of it is received by detector. The scattering behavior of human tissue cause extra traveling of NIR light photons than source-detector separation on scalp. A parameter, namely the differential path length factor (DPF), is multiplied by the actual distance to account for the additional distance traveled by NIR light. The DPF value varies for different wavelengths of NIRS light and as well as for subject's age (Duncan et al., 1995; Kohl et al., 1998). Initially, it was common practice to use DPF values between three to six (Delpy et al., 1988; Duncan et al., 1995). The DPF can vary for different tissue properties and structures as well (Kamran et al., 2016). Jasdzewski et al. (2003) analyzed the effects of DPF on HRF characteristics for a particular set of values ranging from three to twelve. Comprehensive analysis, however, is still required in this field. In addition to scattering behavior, the signal drift in fNIRS data has strong relation with HRF features (Shah and Seghouane, 2014; Metz et al., 2015). As the depth of initial dip is very small as compared to main peak, thus even if small drift is present in the data, the initial dip could not be found and in case where initial dip features are selected for BCI algorithm, the false decision could be expected and possible. A typical methodology for correction of drift is de-trending (Herrera-Vega et al., 2017). High-pass filtering is another fruitful way of removing low frequency drift in HbO signal (Cui et al., 2010). NIRS-SPM, a freely available fNIRS-analysis software package developed by Ye et al. (2009), has proposed wavelet-based de-trending algorithms to abrogate baseline drift. The high computation cost of wavelet-based de-trending algorithms could possibly be abate by utilizing linear de-trending filters (Yin et al., 2015).

**Figure 1:**
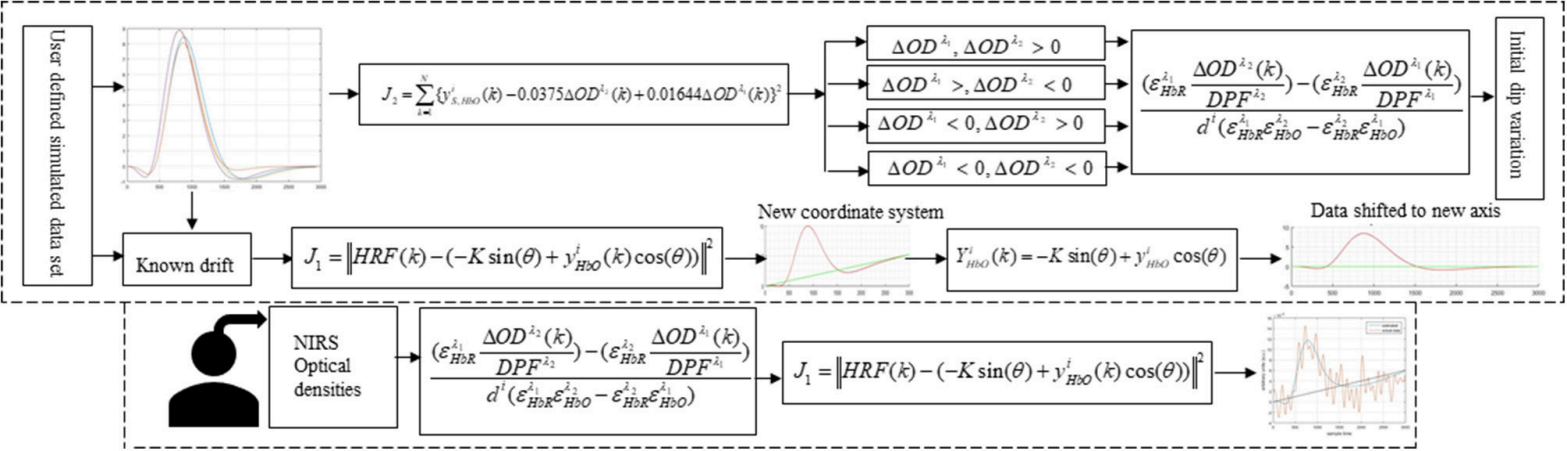
Schematic of algorithm.

In this study, early de-oxygenation of the fNIRS signal was investigated in detail. To that end, the initial dip's existence, estimation, and appearance according to DPF variation and data drift were explored. Based on the results, a scheme for drift estimation was developed and is herein proposed. Additionally, a neuro-activation model and iterative-optimization-based solution were developed, the results for which were evaluated and summarized on the basis of a comprehensive analysis. Additionally, verification analyses for simulated data is performed. Later, real human brain signals were acquired from six healthy subjects and their experiment-related-HRF were estimated using proposed algorithm. A generic overview of the study is presented in Figure 1.

## Theory

### Instrumentation

The experiments were performed with continuous wave NIRS system (DYNOT: Dynamic Near-Infrared Optical Tomography developed by NIRx Medical Technologies, Brookly, NewYork). The sampling rate of the instrument was 1.81 Hz. The data was re-sampled at 100 Hz (MATLAB built-in-command *resample*) for further processing and analysis. It has 32optodes that can be utilized as source or detectors depending upon configuration. The study was approved by local review board of Pusan National University.

### NIRS Data Pre-processing

Concentration changes of HbO and HbR are directly related to the CBF in a particular brain region. These chromophores in blood hemoglobin can be estimated through the relation between incident and attenuated NIR light intensity that is described in the modified Beer-Lambert law (MBBL). The resultant mathematical expressions are described in Delpy et al. (1988), Maikala (2010), and Kamran and Hong (2014). Assuming two wavelengths λ_1_ andλ_2_, the expressions for HbO and HbR concentration changes according to the MBLL can be written as

(1) $$\begin{array}{l} \Delta HbO^{i}\left(k\right)=\frac{\left(\varepsilon_{HbR}^{\lambda_{1}}\frac{\Delta OD^{\lambda_{2}}\left(k\right)}{DPF^{\lambda_{2}}}\right)-\left(\varepsilon_{HbR}^{\lambda 2}\frac{\Delta OD^{\lambda_{1}}\left(k\right)}{DPF^{\lambda_{1}}}\right)}{l^{i}\left(\varepsilon_{HbR}^{\lambda_{1}}\varepsilon_{HbO}^{\lambda_{2}}-\varepsilon_{HbR}^{\lambda_{2}}\varepsilon_{HbO}^{\lambda_{1}}\right)} \end{array}$$

and

(2) $$\begin{array}{l} \Delta HbR^{i}\left(k\right)=\frac{\left(\varepsilon_{HbO}^{\lambda_{2}}\frac{\Delta OD^{\lambda_{1}}\left(k\right)}{DPF^{\lambda_{1}}}\right)-\left(\varepsilon_{HbO}^{\lambda_{1}}\frac{\Delta OD^{\lambda_{2}}\left(k\right)}{DPF^{\lambda_{2}}}\right)}{l^{i}\left(\varepsilon_{HbR}^{\lambda_{1}}\varepsilon_{HbO}^{\lambda_{2}}-\varepsilon_{HbR}^{\lambda_{2}}\varepsilon_{HbO}^{\lambda_{1}}\right)} \end{array}$$

where Δ*HbO*^*i*^(*k*) and Δ*HbR*^*i*^(*k*)are the relative concentration changes of HbO and HbR, respectively, *k* is the step time, *i* represents the *i*th-channel of the source-detector set, $\varepsilon_{HbO}^{\lambda_{1}}$, $\varepsilon_{HbR}^{\lambda_{1}}$, $\varepsilon_{HbO}^{\lambda_{2}}$ and $\varepsilon_{HbR}^{\lambda_{2}}$ indicate the extinction coefficients (referring to the measure of absorption of light) of HbO and HbR at two different wavelengths, respectively, $\Delta OD^{\lambda_{j}}\left(k\right)$ is the optical density variation at the *k*th-sample time and particular wavelength (*j* = 1, 2), *l*^*i*^is the source-detector separation, and $DPF^{\lambda_{j}}$is the differential path length factor at a particular wavelength (*j* = 1, 2).

### Hemodynamic Response Function

Activation detection from cortical imaging data is nothing but the mapping of a recorded time-series to a function that practically endorses the phenomena of a neural process. A canonical HRF (cHRF) composed of two Gamma functions has frequently been used as an indicator of cortical activity (Friston et al., 1994, 1998). The first Gamma function represents the main response, while the second is responsible for post-stimulus undershoot (Abdelnour and Huppert, 2009). It is evident from previous studies (Menon et al., 1995; Hu et al., 1997; Buxton, 2001; Buxton et al., 2004; Hu and Yacoub, 2012; Yoshino and Kato, 2012) that early deoxygenation is a component of neural activation. Thus, a third Gamma function is required in order to mathematically represent the initial dip in the cHRF model, as

(3) $$\begin{array}{l} h(k)=\left[\frac{k^{\alpha_{1}-1}\beta_{1}^{\alpha_{1}}e^{-\beta_{1}k}}{\Gamma (\alpha_{1})}-\frac{k^{\alpha_{2}-1}\beta_{2}^{\alpha_{2}}e^{-\beta_{2}k}}{6\Gamma (\alpha_{2})}-\frac{k^{\alpha_{3}-1}\beta_{3}^{\alpha_{3}}e^{-\beta_{3}k}}{8\Gamma (\alpha_{3})}\right] \end{array}$$

(4) $$\begin{array}{l} HRF\left(k\right)=a_{0}+a_{1}\left\{h\left(k\right)*u\left(k\right)\right\} \end{array}$$

where*u*is a function describing the onset of stimulus and rest sessions, *h* is the cHRF, α_1_is the delay of the response, α_2_is the delay of the undershoot, α_3_is the delay of the initial dip, β_1_is the dispersion of the response, β_2_is the dispersion of the undershoot, β_3_is the dispersion of the initial dip, and Γ represents the Gamma distribution.

### Rotation of Axis

**Figure 2:**
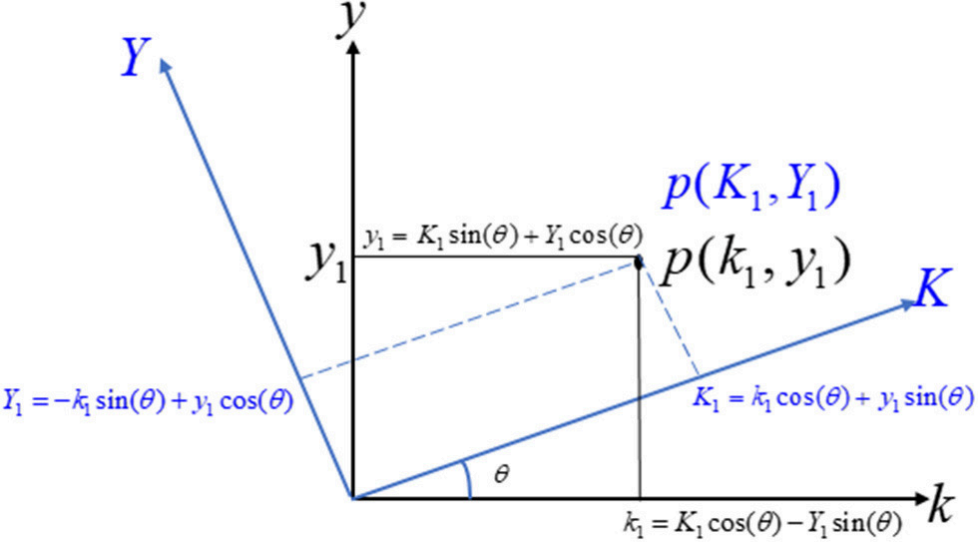
Transformation from one coordinate system to new coordinate system.

Let us consider two coordinate systems, *ky*-axis and *KY*-axis. Consider a point *p*(*k*_1_,*y*_1_) on the *ky*-axis, its representation on the *KY*-axis being *P*(*K*_1_,*Y*_1_), as shown in Figure 2. Further suppose that the rotation angle between the *ky*-axis and the *KY*-axis is θ. The relationship between the two coordinate systems can easily be derived as Anton et al. (2010).

(5) $$\begin{array}{l} k=K\cos\left(\theta \right)-Y\sin\left(\theta \right) \end{array}$$

(6) $$\begin{array}{l} y=K\sin\left(\theta \right)+Y\cos\left(\theta \right) \end{array}$$

(7) $$\begin{array}{l} K=k\cos\left(\theta \right)+y\sin\left(\theta \right) \end{array}$$

(8) $$\begin{array}{l} Y=-k\sin\left(\theta \right)+y\cos\left(\theta \right) \end{array}$$

### Activation Model

The optical signal measured by fNIRS is an amalgam of different signals. These amalgams mainly consist of neuronal-activity-related signal depending upon stimulation and other rhythms responsible for different cortical activities related to the healthy functionality of the human body. The neuronal activity signal is the HRF, and other signals include the respiratory signal, the heart-rate signal, Mayer waves, and noise (Prince et al., 2003; Abdelnour and Huppert, 2009). Thus, the simplest optical brain model can be mathematically represented as

(9) $$\begin{array}{l} y_{HbO}^{i}\begin{array}{l} \left(k)=HRF(k)+\varepsilon^{i}(k\right) \end{array} \end{array}$$

where $y_{HbO}^{i}\left(k\right)$ is the measured HbO concentration change and ε^*i*^(*k*) is the noise term at the *k*th-sample time.

Let us suppose that the measured data has a drift of angleθ ; using equations (7) and (8), re-evaluating equation (9), we obtain

(10) $$\begin{array}{l} K=k\cos\left(\theta \right)+y_{HbO}^{i}\left(k\right)\sin\left(\theta \right) \end{array}$$

(11) $$\begin{array}{l} Y_{HbO}^{i}\left(k\right)=-k\sin\left(\theta \right)+y_{HbO}^{i}\left(k\right)\cos\left(\theta \right) \end{array}$$

Now let us define a cost function that formulates the above problem into an optimization problem as follows:

(12) $$\begin{array}{l} J_{1}={\left||HRF\left(k\right)-\left(-K\sin\left(\theta \right)+y_{HbO}^{i}\left(k\right)\cos\left(\theta \right)\right)|\right|}^{2}.\\ \min J_{1}\left(\theta ,\alpha_{1},\alpha_{2},\alpha_{3},\beta_{1},\beta_{2},\beta_{3},a_{o},a_{1}\right) \end{array}$$

The proposed activation model could be estimated by solving equation (12) for the free parameters inmin*J*_1_(θ, α_1_, α_2_, α_3_, β_1_, β_2_, β_3_, *a*_*o*_, *a*_1_). Thus, the optimal values of free parameters $\left(\theta^{*},\alpha_{1}^{*},\alpha_{2}^{*},\alpha_{3}^{*},\beta_{1}^{*},\beta_{2}^{*},\beta_{3}^{*},a_{0}^{*},a_{1}^{*}\right)$ are estimated using an improved version of a simplex algorithm [Nelder-Mead Simplex (NMSM)]. The NMSM is an iterative optimization method for complex problems. It has three main steps for searching optimal solutions: ordering, centroid, and transformation. Details on the NMSM are available in the literature (Lagarias et al., 1998; Luersen and Le Riche, 2004; Haftka and Gürdal, 2012; Kamran et al., 2015). The most important step to evaluate the upper bound vertex of the cost function is achieved through four sub steps those are reflection, expansion, contraction, and shrinkage (Lagarias et al., 1998; Kamran et al., 2015). The mathematical formulation for Reflection:$x_{r}=\bar{x}+\delta_{1}\left(x_{h}-\bar{x}\right)$,

Expansion:$x_{e}=\bar{x}+\delta_{2}\left(x_{r}-\bar{x}\right)$, Contraction:$x_{c}=\bar{x}+\delta_{3}\left(x_{h}-\bar{x}\right)$, and Shrinkage:$x_{e}=\bar{x}+\delta_{4}\left(x_{l}-x_{i}\right);i=0,1,\ldots ,n$, whereδ_1_, δ_2_, δ_3_, and δ_4_are coefficients of reflection, expansion, contraction and shrinkage, respectively. The typical values of these coefficients have been chosen as 1, 2, 0.5, and 0.5, respectively (Lagarias et al., 1998 and Luersen and Le Riche, 2004).

### Experimental Procedure and Paradigm

**Figure 3:**
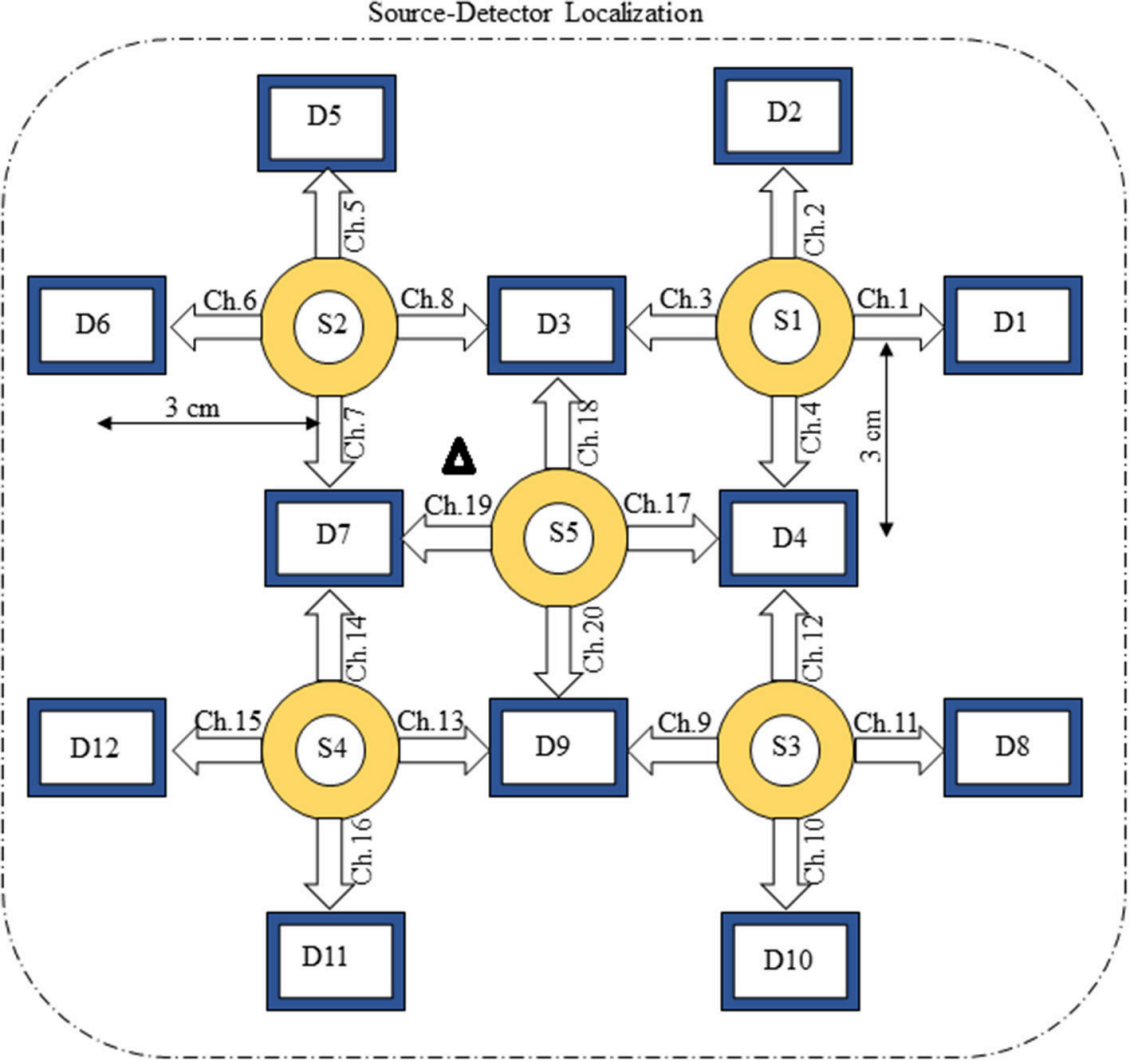
Source-detector localization.

The present experiment was performed on six healthy human subjects of 28 ± 7 years mean age. The experiment was conducted in accordance with the latest version of the Declaration of Helsinki. None of the subjects had a history of any neuronal disorder. All the subjects were university students and were briefed on the experimental procedure. The written consent of each participant was collected before experimentation. The experiment was performed in a shielded room. The subjects were advised to avoid un-necessary movements to reduce artifacts in the measured signal. The subjects were seated on a comfortable chair with load of fNIRS fibers on a hook provided with DYNOT-232 instrument. The experiment included an initial rest of 5 s and a finger-tapping task of 1 s followed by an additional 29 s of rest session. The data recorded for initial rest session was truncated before further processing. The task and rest session instructions were delivered via a monitor placed 100 cm from the subject. The source-detector separation was ~3 cm. Figure 3 presents the source-detector localization scheme with reference position (C3 on 10–20 system) is marked as black triangle.

### Effect of DPF on Initial-Dip

It is mathematically evident in equations (1)- (2) that the DPF has a strong relation to the HRF while optical densities are converted into HbO and HbR concentration changes. A change in the DPF value can alter HRF shape and features. The magnitude of an initial dips' peak is much shorter than the peak of the main response. Therefore, a change in peak value could be a possible factor leading to doubt of the existence of the initial dip. Thus, it is crucial to determine the impact of DPF variation on initial dip. To that end, simulated data sets with different characteristics of HRF were generated. The main peak, post-stimulus undershoot and initial dip of each data set has been shown in **Figure 5**. Later, physiological noises and Gaussian noise were added to the data according to the methodology introduced in Prince et al. (2003) and Kamran et al. (2015). The simulated data sets were fed into equation (1) with known values of $DPF^{\lambda_{1}}$ and $DPF^{\lambda_{2}}$ as well as extinction coefficients and source-detector separation to define the cost function as

(13) $$\begin{array}{l} J_{2}=\overset{N}{\underset{k=1}{\sum }}{\left\{y_{S,HbO}^{i}\left(k\right)-0.0375\Delta OD^{\lambda_{1}}\left(k\right)+0.01644\Delta OD^{\lambda_{2}}\left(k\right)\right\}}^{2}, \end{array}$$

where $y_{S,HbO}^{i}\left(k\right)$ represents the *i*th simulated data set at the *k*th sample time. The above equation was solved for optical densities $\Delta OD^{\lambda_{1}}$ and $\Delta OD^{\lambda_{2}}$to minimize *J*_2_. It has infinitely many solutions for $\Delta OD^{\lambda_{1}}$ and $\Delta OD^{\lambda_{2}}$. Therefore, for further analysis, the solutions were categorized into four different classes: (1):$\Delta OD^{\lambda_{1}}>0,\Delta OD^{\lambda_{2}}>0$, (2):$\Delta OD^{\lambda_{1}}>0,\Delta OD^{\lambda_{2}}<0$,(3):$\Delta OD^{\lambda_{1}}<0,\Delta OD^{\lambda_{2}}>0$, and (4):$\Delta OD^{\lambda_{1}}<0,\Delta OD^{\lambda_{2}}<0$. One solution of equation (13) in each case was fed into the equation below to regenerate the actual signal and verify the obtained solution:

(14) $$\begin{array}{l} y_{S,HbO}^{i}\left(k\right)=0.2170\frac{\Delta OD^{\lambda_{2}}\left(k\right)}{DPF^{\lambda_{2}}}-0.1015\frac{\Delta OD^{\lambda_{1}}\left(k\right)}{DPF^{\lambda_{1}}}. \end{array}$$

In the first step, the values of $DPF^{\lambda_{1}}$ and $DPF^{\lambda_{2}}$ that had been selected during the iterative optimization of *J*_2_ were used. Later, different values of $DPF^{\lambda_{1}}$ and $DPF^{\lambda_{2}}$were used to analyze the effects of those factors on initial dip. The proposed algorithm has two stopping criterions, either could stop the iteration which fulfilled first. The first one is the absolute error in estimated and actual data sets in each iteration and if it is less than a user defined value the iteration stops and consider this as optimal solution. The second one is if the value of solution repeats at succeeding iterations.

## Results

**Figure 4:**
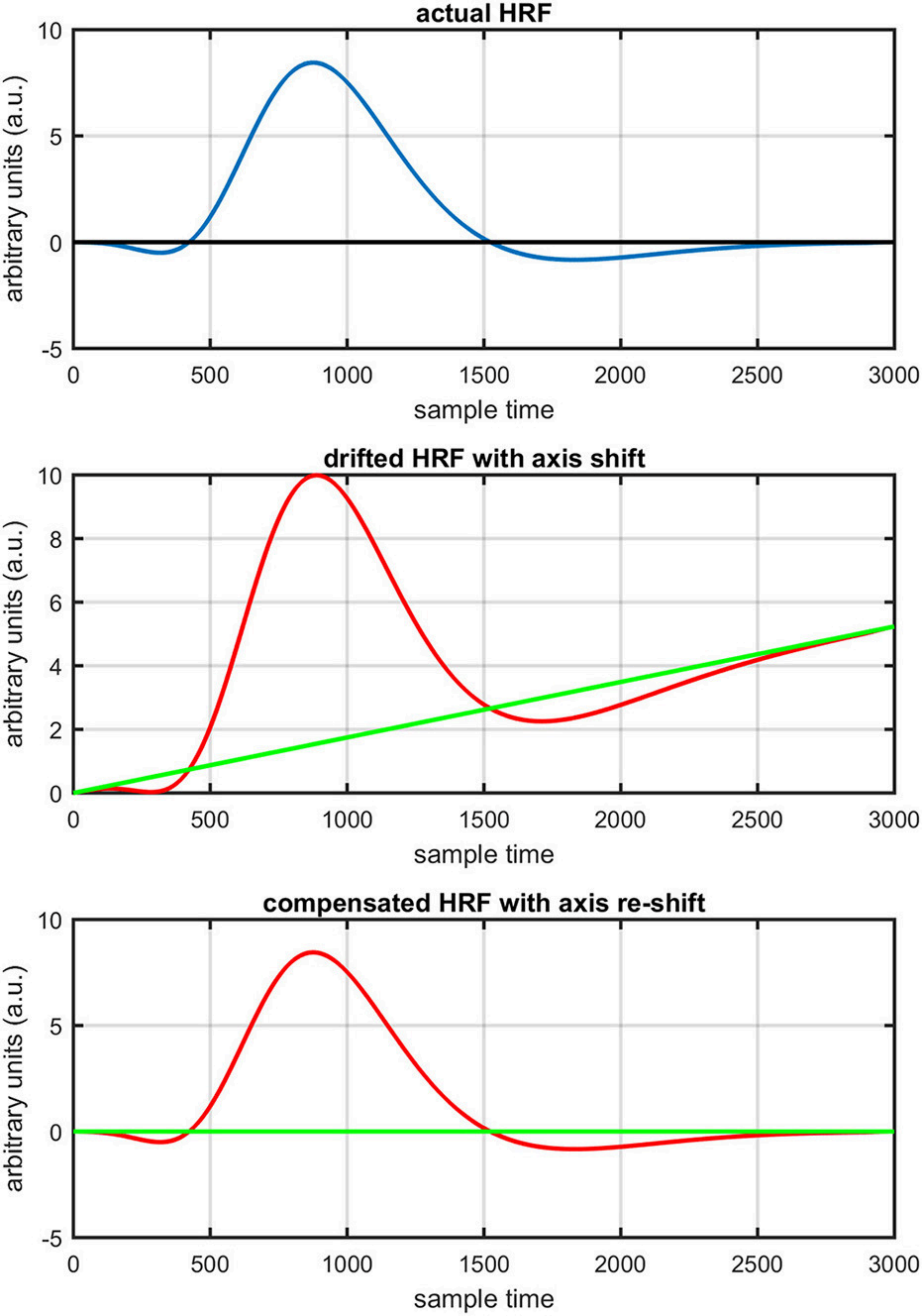
Concept of axis re-shift: actual HRF with standard coordinate system (top plot), data drift and new estimated coordinate system (middle plot), data view in new estimated coordinate system (bottom plot).

**Figure 5:**
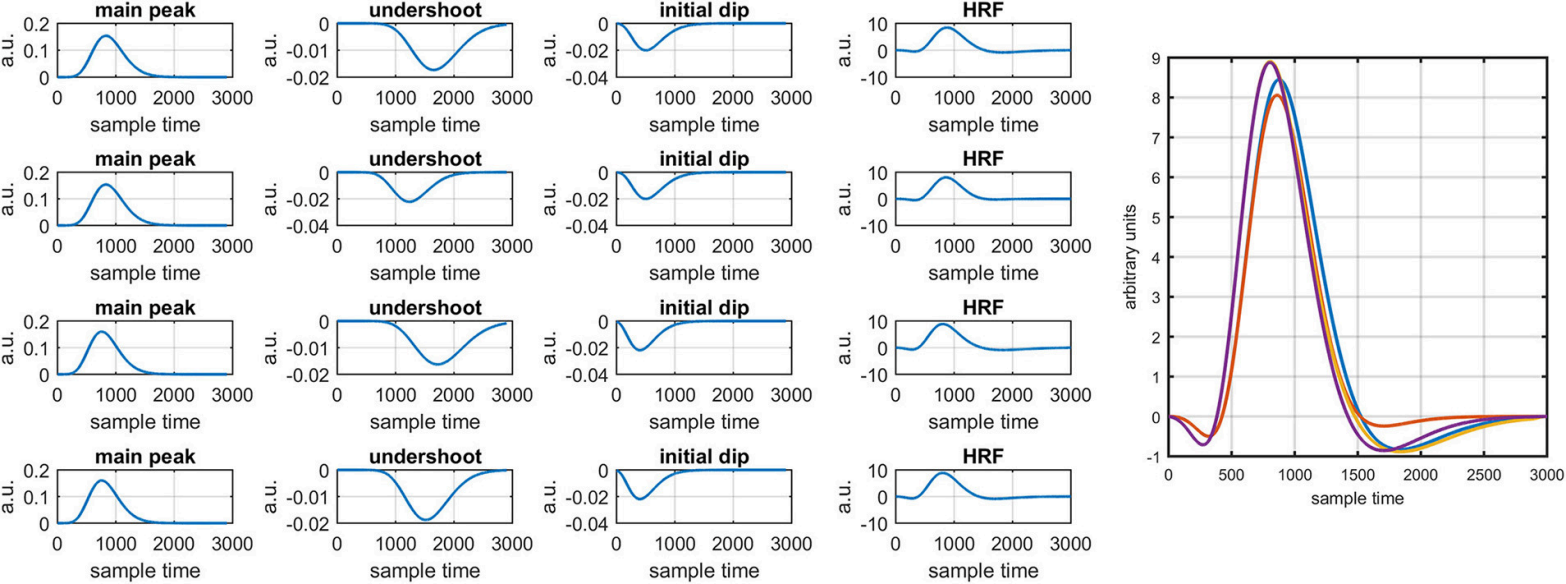
Simulated HRFs and their corresponding main peaks, initial dips and post-stimulus undershoots.

Figure 1 provides a schematic of the proposed algorithm. It has two main parts separated by dotted blocks. The upper larger block shows the analysis and verification on synthetic data sets. It displays the generated HRF with different features, DPF in four different solution categories and estimation of drift in the data. The smaller block represents the optimal-neuro-activation model and its solution by using proposed methodology. Figure 2 displays the concept of coordinate-system transformation. Figure 3 provides the source-detector localization scheme. Figure 4 shows the concept and estimation of drift in the data. The upper plot of Figure 4 shows HRF and middle plot displays a drifted version of it with new coordinate system estimated through proposed algorithm. The bottom plot of Figure 4 presents the visualization of HRF in new coordinate system. Figure 5 displays the simulated data sets with their different characteristics of main peak, post-stimulus undershoot and initial dip.

**Figure 6:**
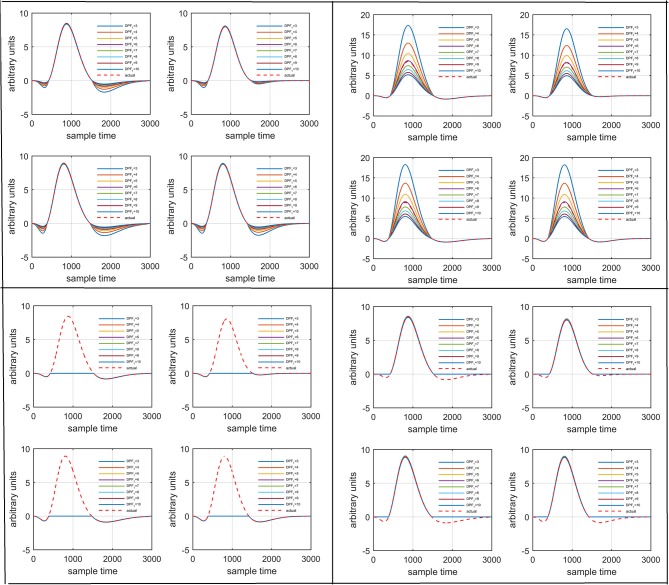
Results for variation of$DPF^{\lambda_{1}}$: Case 1 (top left), Case 2 (top right), Case 3 (bottom left), and Case 4 (bottom right) for four different solution categories, respectively.

**Figure 7:**
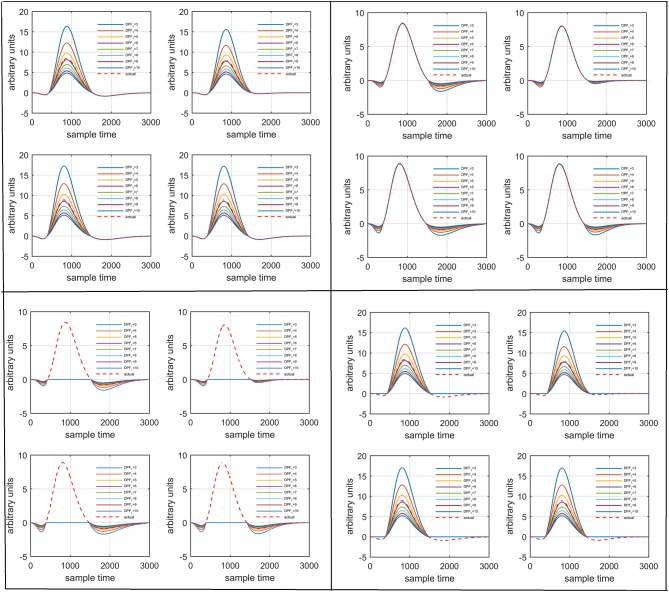
Results for variation of$DPF^{\lambda_{2}}$: Case 1 (top left), Case 2 (top right), Case 3 (bottom left), and Case 4 (bottom right) for four different solution categories, respectively.

**Figure 8:**
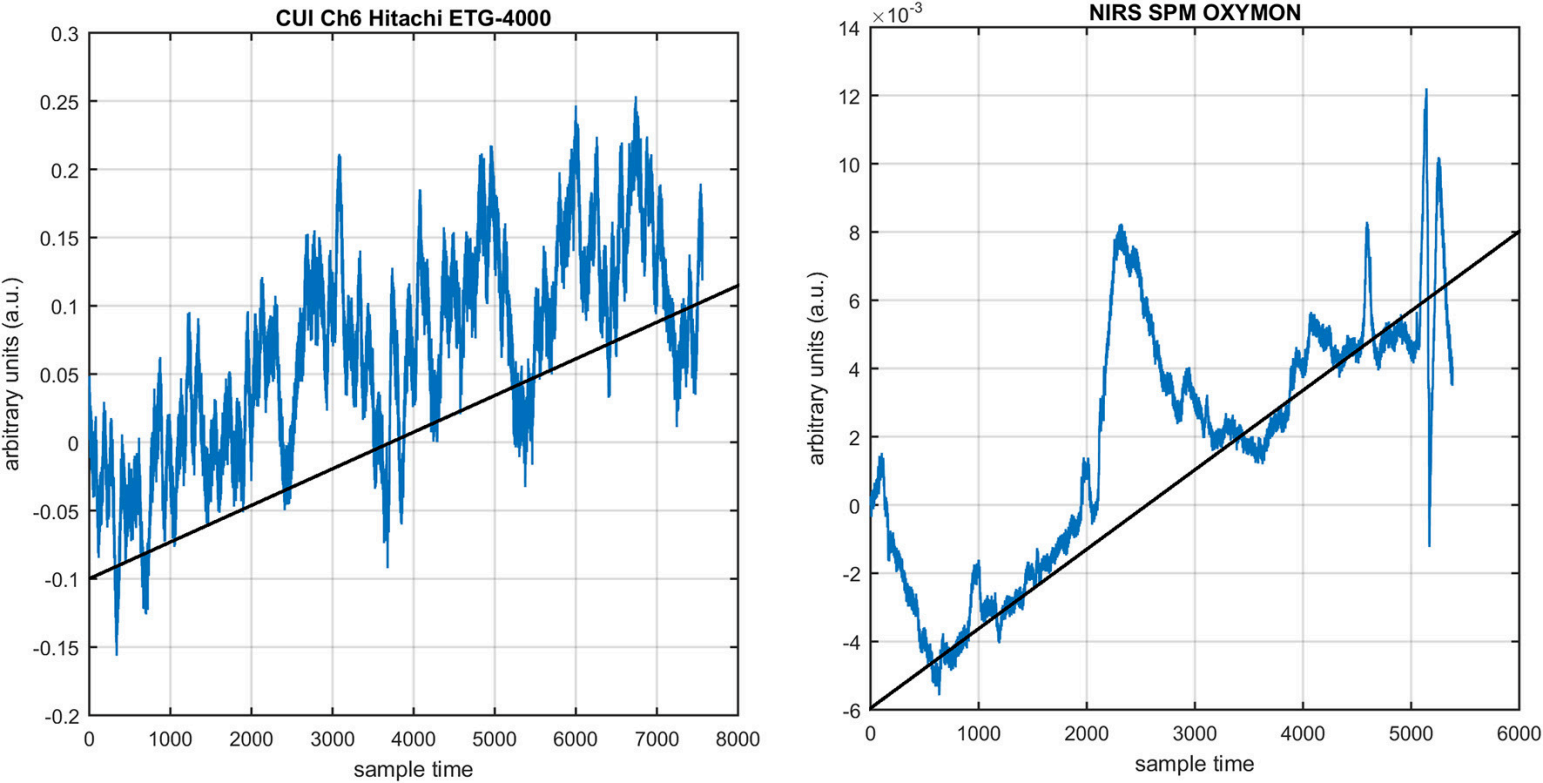
NIRS data drift in freely available data sets: NIRS-SPM, OXYMON (right) and Hitachi ETG-400 (left).

**Figure 9:**
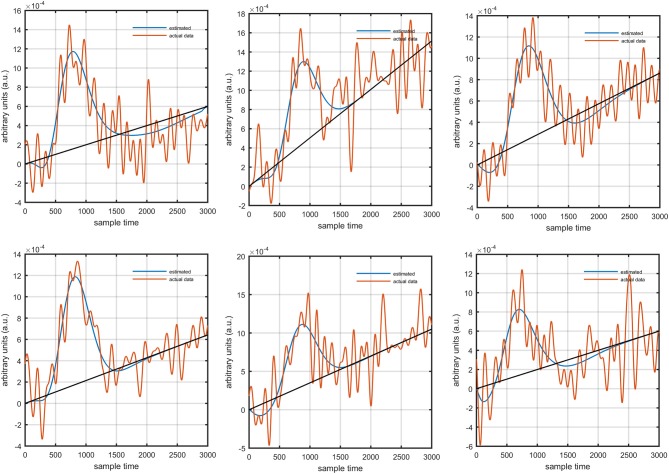
Estimation of best HRF fit and drift using proposed algorithm.

**Figure 10:**
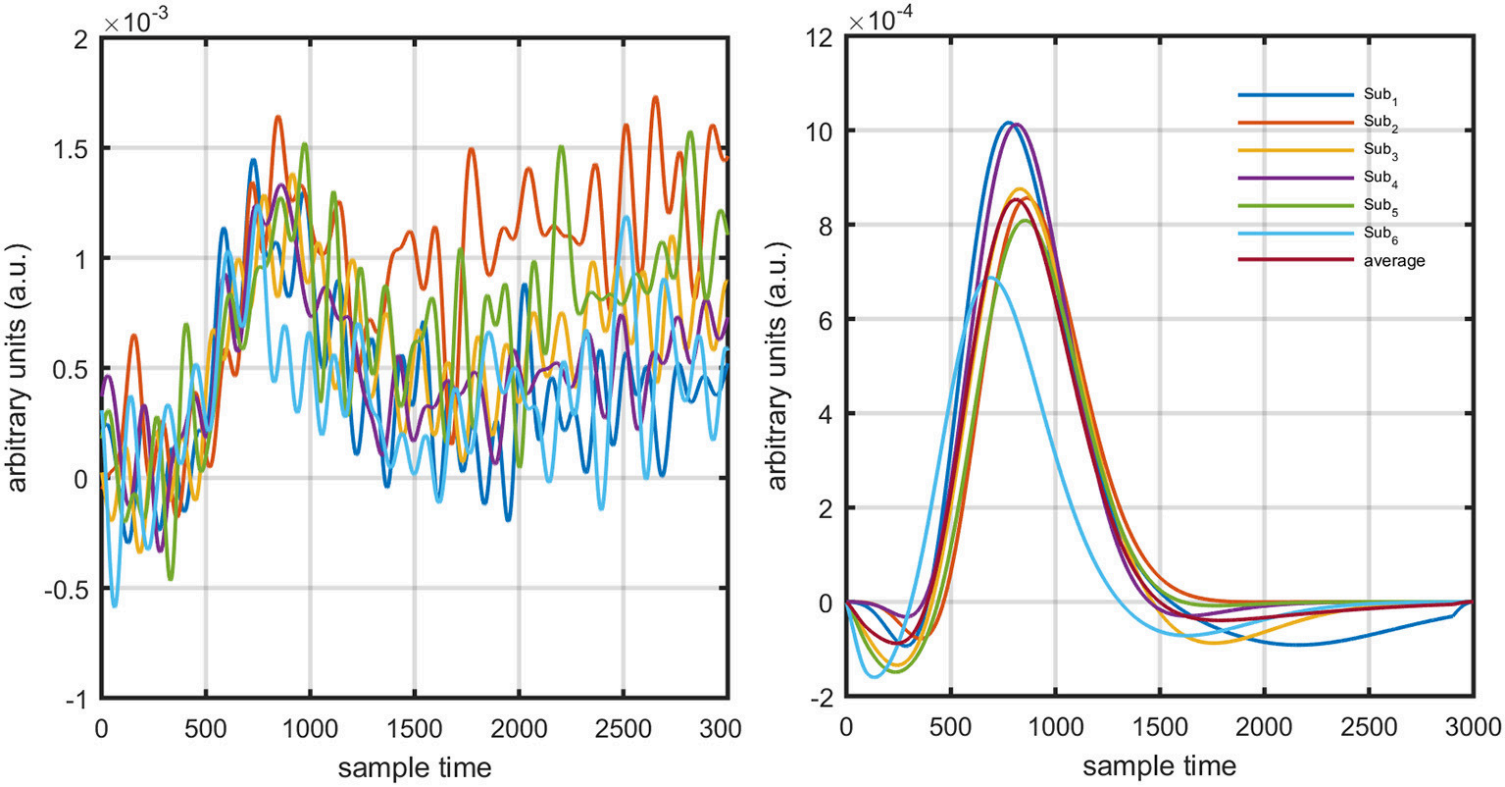
NIRS data sets and corresponding HRF fits using proposed minimization algorithm.

**Figure 11:**
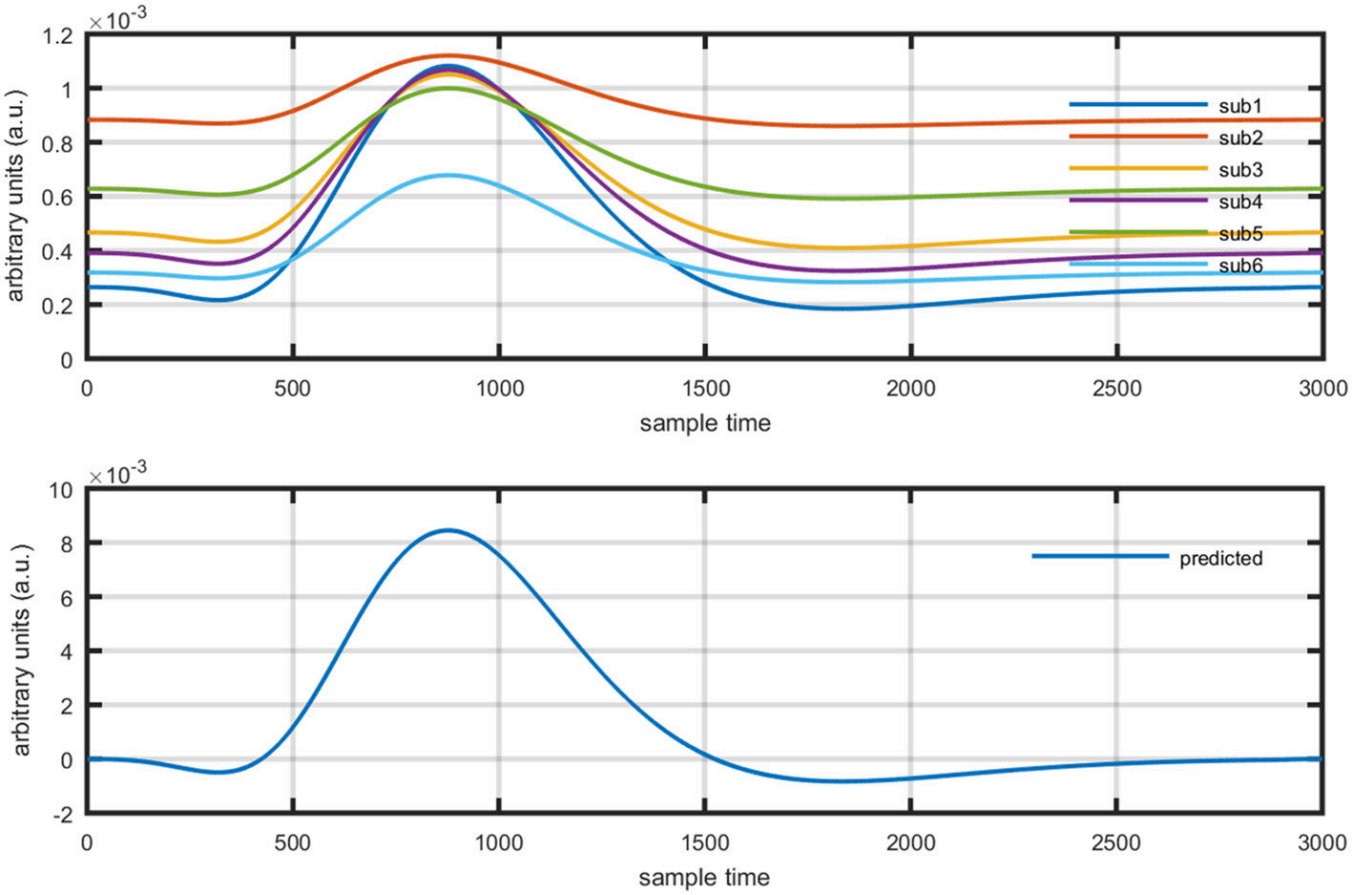
Results using GLM methodology: estimated HRF in NIRS signal of six subjects (top plot) and predicted HRF (bottom plot).

**Figure 12:**
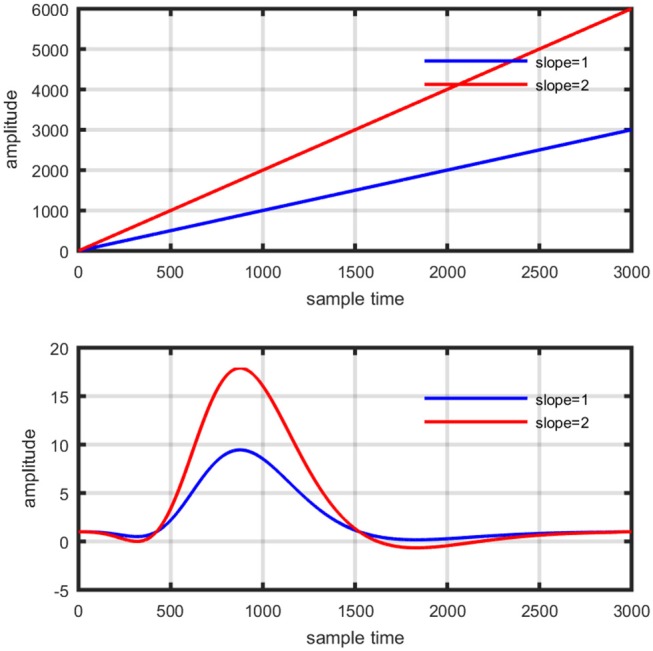
Concept of drift in GLM: slope variation on straight line (top plot), and activity strength variation on HRF (bottom plot).

**Figure 13:**
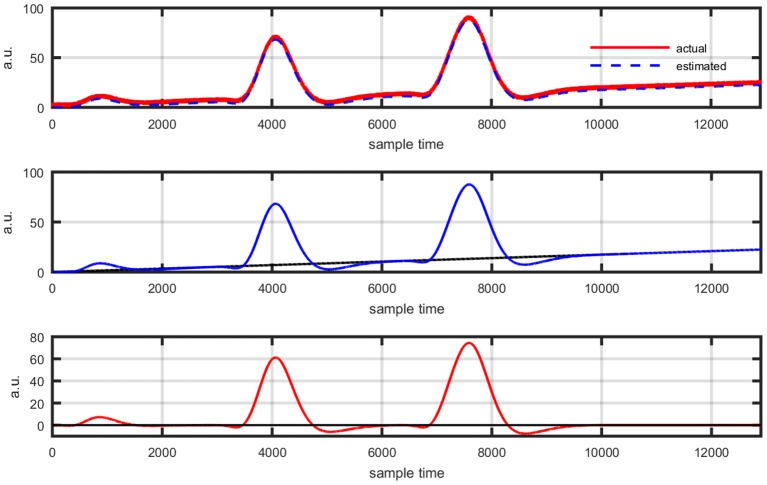
Event-related experimental paradigm's results: actual data and its best possible fit using proposed scheme (top plot), estimation of new coordinate system corresponding to evaluated drift value (middle plot), and data view in new coordinate system (bottom plot).

Several studies in the literature have determined that the DPF is a possible cause of misleading results (Duncan et al., 1995, 1996; Jasdzewski et al., 2003). Therefore, comprehensive analysis of the effects of $DPF^{\lambda_{1}}$ and $DPF^{\lambda_{2}}$on initial dip is essential. In this study, several simulated data sets were generated with different shapes and properties/features of HRF. Then, the cost function *J*_2_ was minimized. The solutions were categorized into four cases. In each case, first, the value of $DPF^{\lambda_{1}}$was fixed and that of $DPF^{\lambda_{2}}$ was varied. Conversely, in the next step, the value of$DPF^{\lambda_{2}}$was fixed and that of $DPF^{\lambda_{1}}$ was varied. Case 1: Both optical densities were constrained to be positive. In this case, as $DPF^{\lambda_{1}}$was changed from a lower value, 3, to a higher value, 10, a gradual decrease in initial dip was observed, and negligible initial-dip change was observed as $DPF^{\lambda_{2}}$was varied. Case 2: Both optical densities were constrained to be negative. This case showed a very small change in initial dip as $DPF^{\lambda_{1}}$was varied, whereas the initial dip decreased with variation of$DPF^{\lambda_{2}}$. Case 3: The optical density related to wavelength λ_1_ was constrained to be positive while the one related to wavelength λ_2_ was constrained to be negative. In this case, there was negligible change in initial dip as $DPF^{\lambda_{1}}$was increased, and the initial dip decreased as$DPF^{\lambda_{2}}$was varied. Case 4: The optical density related to wavelengthλ_1_was constrained to be negative while the one related to wavelength λ_2_ was constrained to be positive. In this case, no effect on initial dip was observed for either the $DPF^{\lambda_{1}}$or $DPF^{\lambda_{2}}$variations. One of the main reasons for the result in this case was that negative output was not possible. Figure 6 depicts the effects of variation of $DPF^{\lambda_{1}}$for four different cases. Figure 7 depicts the effects of variation of$DPF^{\lambda_{2}}$ on for four different solution categories. Figure 8 plots the drift in real data sets freely available with fNIRS software packages [NIRS-SPM developed by Ye et al. (2009), Cui et al. (2010)] with their respective drifts. Figure 9 plots the estimation of drift in real data sets with best possible fitting of the free HRF to the fNIRS measured signal. Figure 10 shows the HbO signals measured by DYNOT-232 from six healthy subjects and corresponding HRFs estimated through proposed algorithm. Figure 11 depicts the estimation of corresponding HRF among subjects using GLM-methodology with designed HRF shown in bottom plot of Figure 11. The concept of drift in GLM is presented in Figure 12. Figure 13 shows the results of proposed scheme related to event-related task.

## Discussion

Optical spectroscopy is an emerging technique that measures spectral distributions of light energy to infer NIR light interaction with human skin, scalp, tissues, and blood hemoglobin, among others. Neuro-activation-dependent oxygen demand causes changes in the concentration of HbO and HbR in a particular brain area responsible for specific task (Yacoub and Hu, 2001; Yoshino and Kato, 2012). This neuronal activity accompanies early de-oxygenation i.e., HbR increase before increase in oxygenated hemoglobin (Hu and Yacoub, 2012). The CBF increases much more than cerebral metabolic rate and hence oxygen extraction fraction (E) decreases with activation (Buxton et al., 2004). The ability to measure the spectroscopic information with NIRS allows one to characterize changes in HbO and HbR separately which results in less ambiguous analysis of activity induced volume and metabolic changes than Blood Oxygen Level Dependent (BOLD) in fMRI (Jasdzewski et al., 2003). On the other hand, the information observed via fNIRS measurement can be affected by several factors, leading thereby to misleading results. Several studies in the field of fNIRS have documented the existence of the initial dip (Jasdzewski et al., 2003; Buxton et al., 2004; Akiyama et al., 2006; Hu and Yacoub, 2012; Yoshino and Kato, 2012), though such dip remains a controversial issue. Therefore, it is necessary to uncover any factor that can affect the existence of initial dip.

The first and most basic factor is the slow drift in HbO signals within fNIRS data. It is important to mention here that drift does not exist in every channel and in every experiment of fNIRS. But it is observed in some of channels during experiments. Figure 8 displays the drift in fNIRS time-series observed in freely available online data sets of fNIRS software packages, [NIRS-SPM (Ye et al., 2009) and (Cui et al., 2010)]. It is evident that if a new coordinate system is defined at a particular angle (shown in Figure 8) estimated with the proposed algorithm, the data can be visualized in that system for correct analysis and overall ease of approach. The drift in the data can affect the existence/estimation of the initial dip. Therefore, the proposed estimation model determines, in the same computations, not only the drift but also the best fit of free HRF.

It is common practice to apply low pass filter and de-trending algorithms to get rid of physiological noises and drift in observed optical data (Abdelnour and Huppert, 2009; Ye et al., 2009; Cui et al., 2010; Shah and Seghouane, 2014; Metz et al., 2015; Yin et al., 2015; Herrera-Vega et al., 2017). As for as physiological noises are concerned, most frequently used filtering range is with cut-off frequency of 0.5 Hz low pass filter and 0.01 for high pass filter to remove these unnecessary signals. In contrast with existing methodologies, the proposed algorithm estimates the drift in the data with best possible fit (if exist) applying node optimization. This is very important point because a slight error in mismatch of particular trend can cause existence/disappearance of initial dip. The drift is found and estimated in single trial and multi-session experimental paradigms using proposed methodology. The authors did not observe mix/multi-trending behavior in fNIRS signal. However, a running correlation is found at each sample time between measured data and its fit. If the running correlation is less than (say 90% of the peak value), the remaining signal could be re-optimized using proposed methodology to predict another trend in the same dataset.

Biological tissues are highly scattered medium of NIR light and consequently Beer-Lambert law expression does not hold as path length of the light is much more longer than physical separation between NIR light source and receiver (Kohl et al., 1998). Thus, it was common practice in past to introduce DPF value between three and six for compensation of additional distance traveled by NIR light (Delpy et al., 1988; Duncan et al., 1995). Jasdzewski et al. (2003) reported the differences in HR to motor and visual paradigms using fNIRS. They additionally analyzed the effects of the variation of DPF upon HRF. Three set of combinations of $DPF^{\lambda_{1}}$ and $DPF^{\lambda_{2}}$were chosen for analysis i.e., 3 and 6, 6 and 6, and 12 and 6. In contrast to available literature, the DPF is varied between three and ten with increment of unity. It is found that DPF can affect the features of initial dip and at worst it could be a possible factor of initial dip disappearance as described by existing literature (Jasdzewski et al., 2003). In addition to this, it is observed that the combination of polarity of optical densities has major role to variate initial dip dependence upon DPF. Therefore, four different cases are presented here analyzing a particular example of data set. The results show that the variation in DPF can affect the peak value of initial dip, post-stimulus undershoot and main response. Scholkmann and Wolf (2013) summarized an important factor that actually cause the variation of DPF among other factors i.e., age of subject. They developed a useful equation that can be used to determine DPF value for a subject of particular age. The authors opinion is that not only age, different brain areas of same age could have different scattering patterns that can cause light photons to travel an extra distance which needs to be catered. But still there is no such algorithm.

The general linear model (GLM) was frequently applied methodology for the analysis of fMRI time series measured by characterizing diamagnetic and paramagnetic behavior of hemoglobin chromophores (Ye et al., 2009). It was developed by Prof. C. J. Friston and is part of statistical parameter mapping tool (Friston et al., 1994) and has also been applied to fNIRS signal analysis using block and event related paradigms (Abdelnour and Huppert, 2009; Ye et al., 2009; Kamran and Hong, 2014). The application of GLM to fNIRS data has shown enlightening results, but still it has certain limitations. The crux of GLM is to split and represent measured time series as a linear combination of predicted hemodynamic response function (pHRF) and a base line, usually known as basis set (Çiftçi et al., 2008; Ye et al., 2009; Kamran and Hong, 2014). The weights of regressors can be estimated by applying least square fit and mismatch error is considered as noise (Zhang et al., 2013). However, in case of fNIRS, it entails an additional challenge due to various factors causing variability in the measured optical time series. Physiological noises (cardiac beat, respiratory rhythm, and low frequency Mayer waves among others) are major contributor that formulate a mixture with neuronal related concentration changes of HBO and HbR in optical data (Prince et al., 2003; Abdelnour and Huppert, 2009; Kamran and Hong, 2014). The variants of GLM are mostly focused for filtering the data from physiological noises e.g., (Hu et al., 2010) proposed GLM by adding three regressors of cosine series to estimate particular sinusoidal signal. Similarly, (Kamran and Hong, 2014) have proposed ARAM model with exogenous input to explore existence of respiratory rhythm, hear beat related signal, and Mayer waves. In addition to this, Trial-to-trial variability in fNIRS data has been reported under various experimental paradigms (Holper et al., 2012). Thus, a non-linear optimization modeling algorithm can estimate the neuro-activation-dependent hemoglobin concentration changes more efficiently and accurately. Let us suppose a neuro-activation model consist of a basis set of GLM formulation as follows,

(15) $$\begin{array}{l} y_{obs}=\beta_{1}y_{pHRF}+\beta_{2} \end{array}$$

Where *y*_*obs*_is the observed HbO concentration change, β_1_is activity strength parameter and β_2_ is a baseline correction. In this model β_1_ can be regarded as slope of a line represented mathematically in equation (15). The increase in β_1_ is responsible for drifting the line from one position as shown in Figure 12 (upper plot). Similarly increase in β_1_ constitute scaling of cHRF (or HRF) as shown in Figure 12 (bottom plot). This parameter is regarded as indirect measure of neuro-activation (Friston et al., 1994; Abdelnour and Huppert, 2009; Ye et al., 2009; Kamran and Hong, 2014). Another limitation of GLM, in case of fNIRS, is the dependency of output upon the cHRF designed and convolved with experimental paradigm, called pHRF. Whatever *y*_*pHRF*_ is designed based upon two/three Gamma functions, the activity strength parameter is the only factor that characterize the existence and shape of neuro-activation. In literature however, different methodologies were proposed to constraint GLM environment that can improve the accuracy and variability (different characteristics and features) in estimation of predicted HRF (pHRF) features for fNIRS (Woolrich et al., 2004; Çiftçi et al., 2008). In proposed methodology, the shape of neuro-activation waveform is free and highly dependent upon observed time series, in contrast with standard GLM.

## Conclusion

In this paper, existence of the initial dip and its appearance as a function of data drift and DPF is explored comprehensively. In addition to this, a novel neuro-activation model that can estimate drift in fNIRS data and determines the best fit of HRF was developed. In contrast with existing methodologies, where predicted HRF fed as a part of estimation model, the HRF shape and features were supposed to be free parameters. Furthermore, drift in fNIRS data could be main factor whose slight estimation error could result in disappearance of initial dip. It is also concluded that DPF can affect the shape of initial dip. Additionally, the effects of activity strength parameters in standard GLM is analyzed.

## Ethics Statement

This study was carried out in accordance with the recommendations of Declaration of Helsinki, Pusan National University, ethical committee. The protocol was approved by the Pusan National University, ethical committee.

## Author Contributions

MK has proposed the methodology to analyze fNIRS data and results, simulations, and wrote the first draft of the manuscript. MN has done literature survey, implementation of methodology, and helped in editing of manuscript before submission. M-YJ has supervised the research.

### Conflict of Interest Statement

The authors declare that the research was conducted in the absence of any commercial or financial relationships that could be construed as a potential conflict of interest.
